# Donor Autonomy and Self-Sacrifice in Living Organ Donation: An Ethical Legal and Psychological Aspects of Transplantation (ELPAT) View

**DOI:** 10.3389/ti.2022.10131

**Published:** 2022-03-21

**Authors:** Nizam Mamode, Kristof Van Assche, Lisa Burnapp, Aisling Courtney, David van Dellen, Mireille Houthoff, Hannah Maple, Greg Moorlock, Frank J. M. F. Dor, Annette Lennerling

**Affiliations:** ^1^ Department of Transplantation, Guys and St Thomas NHS Foundation Trust, London, United Kingdom; ^2^ Research Group Personal Rights and Property Rights, University of Antwerp, Antwerp, Belgium; ^3^ Regional Nephrology and Transplant Unit, Belfast City Hospital, Belfast, United Kingdom; ^4^ Department of Renal and Pancreas Transplantation, Manchester University NHS Foundation Trust, Manchester, United Kingdom; ^5^ Erasmus MC Transplant Institute, Department of Internal Medicine, University Medical Centre, Erasmus University Rotterdam, Rotterdam, Netherlands; ^6^ Warwick Medical School, University of Warwick, Coventry, United Kingdom; ^7^ Imperial College Renal and Transplant Centre, Hammersmith Hospital, Imperial College Healthcare NHS Trust, London, United Kingdom; ^8^ Department of Surgery and Cancer, Imperial College, London, United Kingdom; ^9^ The Transplant Centre, Sahlgrenska University Hospital, Gothenburg, Sweden; ^10^ Institute of Health and Care Sciences, The Sahlgrenska Academy, University of Gothenburg, Gothenburg, Sweden

**Keywords:** risk, kidney, transplantation, living donation, autonomy 2

## Abstract

Clinical teams understandably wish to minimise risks to living kidney donors undergoing surgery, but are often faced with uncertainty about the extent of risk, or donors who wish to proceed despite those risks. Here we explore how these difficult decisions may be approached and consider the conflicts between autonomy and paternalism, the place of self-sacrifice and consideration of risks and benefits. Donor autonomy should be considered as in the context of the depth and strength of feeling, understanding risk and competing influences. Discussion of risks could be improved by using absolute risk, supra-regional MDMs and including the risks to the clinical team as well as the donor. The psychological effects on the donor of poor outcomes for the untransplanted recipient should also be taken into account. There is a lack of detailed data on the risks to the donor who has significant co-morbidities.

## Introduction

**FIGURE 1:**
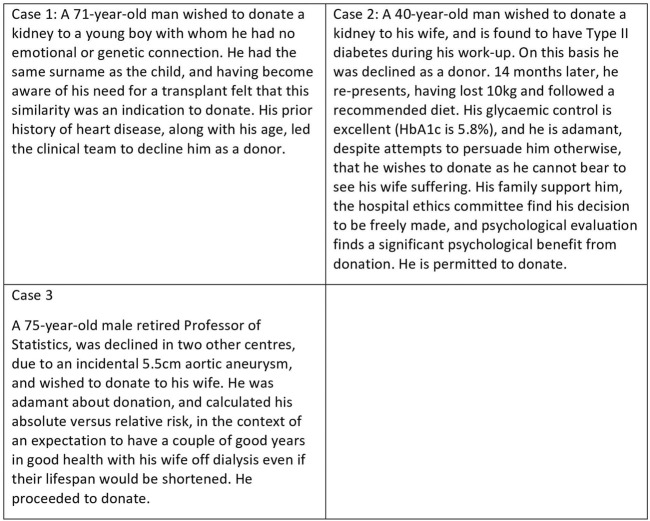
Examples of potentially difficult decisions regarding living donor candidates.

**FIGURE 2:**
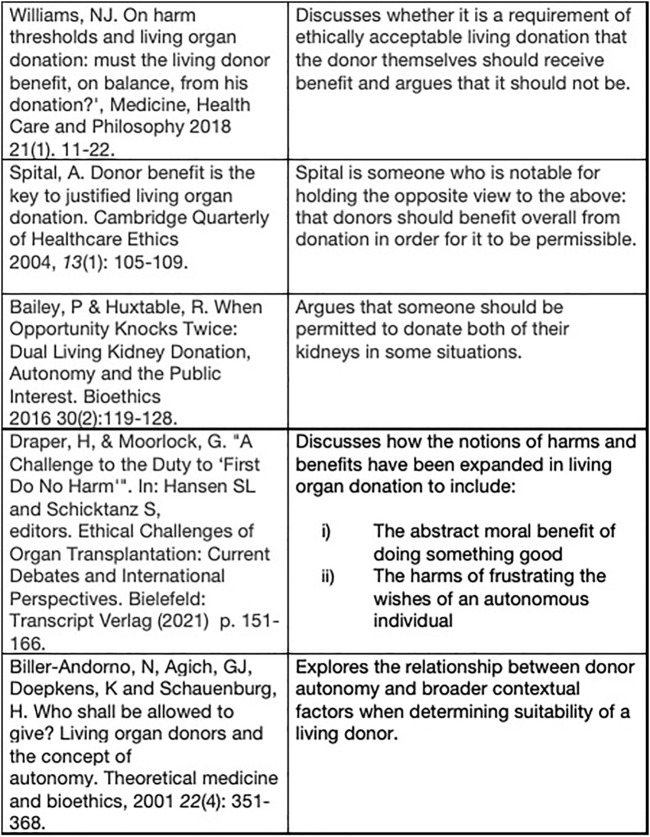
Suggested further reading.

The donation of a solid organ for transplantation by a person who is alive at the time represents a unique event in healthcare, since the donor will gain no physical benefit from undergoing major surgery, which has a low but nevertheless significant rate of major complications and death (1, 2). Living donors are usually highly motivated individuals, whose appetite for risk differs substantially from that of the healthcare team (3). This may lead to conflicts between the clinical team and potential donors-some examples are given in Figure 1. Were the decisions of the clinical teams correct? This article explores the issues raised by these cases and others, and considers the principles which might help to guide decision-making. It is an overview aimed at healthcare professionals, and is not intended to be an in-depth ethical review. Suggestions for further reading are given in Figure 2.

## Autonomy Versus Paternalism

**FIGURE 3:**
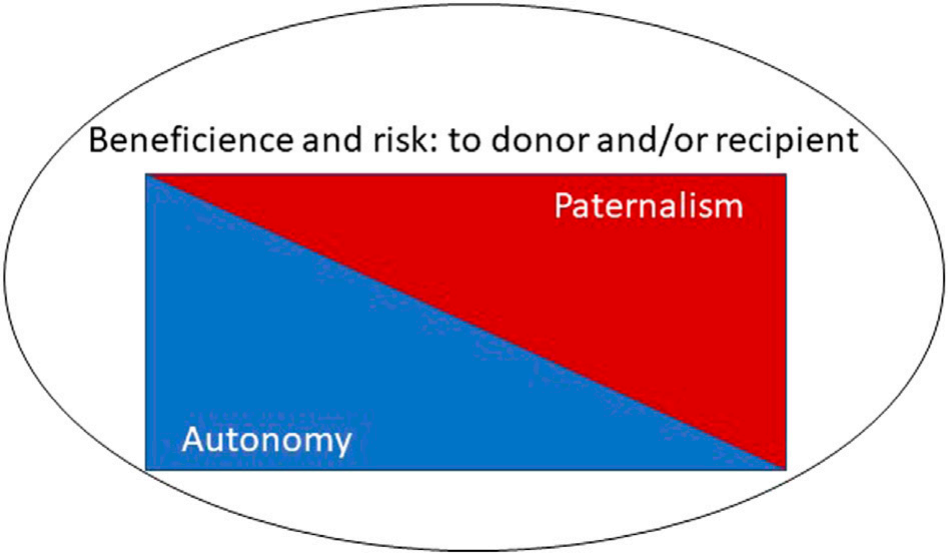
The interplay of potentially conflicting ethical principles.

Although not universally adopted, principlism remains the dominant approach to medical ethics (4), particularly amongst the clinically-orientated. Under a principlist approach, four principles are considered in the determination of whether an intervention is ethically appropriate: autonomy, beneficence, non-maleficence, justice (5). Beauchamp and Childress suggest that each principle should be afforded equal weight, but nonetheless autonomy is often regarded as “first amongst equals” (6). In living kidney donation, beneficence is difficult to both specify and quantify accurately. There is likely to be some psychological benefit (7, 8) but there is clearly no physical benefit of donation itself. Whilst non-maleficence, or more specifically the minimisation of harm is a concomitant aim of donation surgery, some harm is unavoidable, such as the physical harm routinely associated with surgery, and sometimes unanticipated complications occur. Although teams attempt to assess the risk to the donor independently, the benefit to the recipient also plays a part (9), since without this the donation would not be justified (Figure 3). Some have argued for a “donor-centred” approach, where the importance of the emotional benefits to the donor is expanded when considering risks (10).

The clinical team are also agents here and ultimately responsible for decisions to offer donation as an option to an individual: an on-table death of a donor would certainly affect them profoundly, and potentially their programme and others, and hence other patients. But this could perhaps be overcome by having centralisation of high risk cases in dedicated centres or by having surgeons for “high risk” cases in centres, where everyone understood that the risks were higher and appropriate protections were in place, including transparent audit, support for staff, and avoidance of punitive actions in the event of below average outcomes.

It is quite common for clinical teams to adopt a degree of paternalism (11), whereby autonomy is infringed upon to some extent in order to serve a patient’s best interests. Consider, for example, the postoperative patient who would rather not get out of bed, but is essentially cajoled into doing so. In this scenario, it might be considered that the patient’s wish to stay in bed is not strongly held, and that it is heavily in their best interests to mobilise, so beneficence overrules respecting the rather weak autonomous wishes of the patient. It might then seem logical that there is a gradation of potential benefits or harms, which could be weighed against a scale of autonomous desires of increasing strength, rather than simple binary outputs for these potentially competing interests. Considering that there may be effectively different levels of autonomy, related to a degree of understanding and strength of feeling, may help here. Similarly, it might be considered that there is a scale of paternalism, ranging from “weak to strong (12)” or “soft to hard (13).” In practical terms, such an interpretation is necessarily a matter of subjective judgement, but a potentially paternalistic approach might include consideration of the following: how strongly do you feel about donating, and why? Do you have a reasonable understanding of the risks? How likely are you to regret this later? Despite the difficulty in answering these questions, it might be a first step in resolving the conflicts described above.

A key problem in considering the importance of autonomy in medical decision making is the difficulty in the determination of the value that should be accorded to a particular autonomous wish. That is, at what point does an apparently autonomous decision carry sufficient weight to outweigh other considerations (9). This is a key issue when considering decision making in children, who may not yet be considered independent and adults who are incompetent to make any decision, but whose wishes are nevertheless taken into account. Indeed, children not infrequently express a wish to donate to siblings, but in most jurisdictions this would be refused (14, 15). Perhaps a useful ethical approach would be to balance the clinical team’s view of the potential benefits and harms, with the depth and strength of conviction of the individual concerned. One might consider a central aspect of autonomy to be the ability to use relevant information to reason in certain ways and adopt a considered approach (5). Thus, it might be, for example, that an experienced transplant surgeon with non-insulin dependent diabetes who felt strongly that they wished to donate to their spouse could have a reasonable understanding of the risks, and should be allowed to proceed. In clinical practice, a clear understanding of the risks is often given greater validity in terms of decision making; however, it could be argued that neither depth nor strength of conviction are valid reasons for assessing the degree of autonomy. Furthermore, freedom from external pressures beyond the clinical team, for example from family members, is an important consideration in determination of the extent to which a patient’s wishes are truly autonomous.

## Risk Benefit Balance

The risks of donor nephrectomy are mortality 1 in 3,000 and major complications 2–5% (1, 2), while for a living liver donation the mortality rate is 1 in 200 (16). This could mean that a “high risk” kidney donor might still be exposed to less risk than a low risk liver donor. It could be argued that the difference here is the combination of lack of availability of other options and need for urgent surgery in the recipient, since a liver patient might not survive for long without a transplant, while most kidney recipients would have a dialysis option. However, in considering the risk/benefit balance for the donor, the implication must be that the difference is only a psychological one, and not physical-that is, the liver donor has the higher psychological risk of seeing a loved one die, which justifies the higher risk of donation. There can’t be any other moral imperative to expose the donor to higher risks because the stakes are higher for the recipient. The logical extension of this argument suggests, however, that outcomes other than death might have a profound psychological detrimental effect on the potential donor-for example, parental donation to a child who is not thriving on dialysis, or spousal donation where the life of the donor is severely impacted by having an unwell partner (17).

One of the common errors in considering the risks of donation is to focus on relative, rather than absolute, risk. The use of absolute risk has been recommended specifically for living donors (18). A mortality rate of 1 in 1,500 is twice the normal risk but still very low, and lower than for the liver donor. Furthermore, we do not have good data on what the actual risks are in those with co-morbidities, in part because they are usually refused surgery (19). For example, previous myocardial infarction is often an exclusion criterion for kidney donors, yet if successful rehabilitation has taken place, risk factors addressed and cardiac tests are adequate, then it probably does not confer a high absolute risk (20, 21). An alternative approach might be to consider what is an acceptable upper mortality rate, and to permit donation if this threshold is not reached, even if the relative risk is doubled. Clearly challenges would remain in determining this rate, and in assessing individual donors who are below this threshold. There is certainly a need to determine more accurately and objectively the risks to both donor and recipient, in order to make the appropriate decision-just as we may not be aware of the real perioperative risk to a donor conferred by a co-morbidity, data on the risk to the recipient of not proceeding with a living donor transplant at that time is often lacking.

It is also important to consider long term as well as perioperative risk. There is even less data here. For example, the lifetime risk of ESRD after LDN in a 70-year-old man is 0.15% (95% CI 0.05, 0.28), and the relative risk for ESRD from non-insulin dependent diabetes is 3.01 (1.91, 4.74)- the absolute risk would appear to be low, but we have no data on the effect of donation on subsequent ESRD in this scenario (22).

Risk aversion may sometimes vary with specialty; surgeons and nephrologists sometimes have differing appetites for risk. Whilst the multidisciplinary meeting (MDM) or protocols and guidelines may mitigate some of these differences, an exploration of how these operate in practice, and the underlying thought processes could help in smoothing decisions. An emerging literature on cognitive biases and loss aversion, where the fear of a low probability but high loss outcome tends to outweigh potential gains, in decision making indicates an interesting start (23, 24).

Finally, risks apply not only to the potential donor, but to the operating surgeon, the clinical team, and to a national programme, since donor deaths have typically impacted on all of these. One way to mitigate this might be to take national decisions on high-risk cases, in a sense as a supra-regional MDM, which would in part shift some of the risk away for the local team in the same way that local MDM advice shares the risk beyond the operating surgeon. Equity of access is an important principle to consider, since widely differing views may pertain in different centres (18). It is also important to consider the risk to the recipient-a donor who suffers severe complications may lead to considerable distress for the recipient.

## Self-Sacrifice and Heroism

We applaud self-sacrifice in many walks of life-firefighters, military, even sport, such as Formula 1, mountaineering, round the world sailing. Those who take risks to save others, or for glory or money, are often considered heroes. Why is someone who takes a risk as a donor different?

It might be argued that the difference is that they need a clinical team to facilitate their operation- but then many of the others listed above need support from teams. Arguably in these cases there is oversight of risk by another group. For example, a military unit might be ordered to retreat if the risk is too high, or the race director may stop a Grand Prix if rain makes it unsafe. It could be considered that the MDM in each unit provides a similar oversight, but given the potential risks to individual clinicians, and to programmes, of poor outcomes as mentioned above it might be that we are not independent enough. The wide variability in assessment criteria illustrates the difficulty here (19, 25). Nevertheless, if the local clinical team is reluctant to proceed, there is an argument for a second opinion, or for national or regional bodies to make these assessments.

## Extreme Risks

**FIGURE 4:**
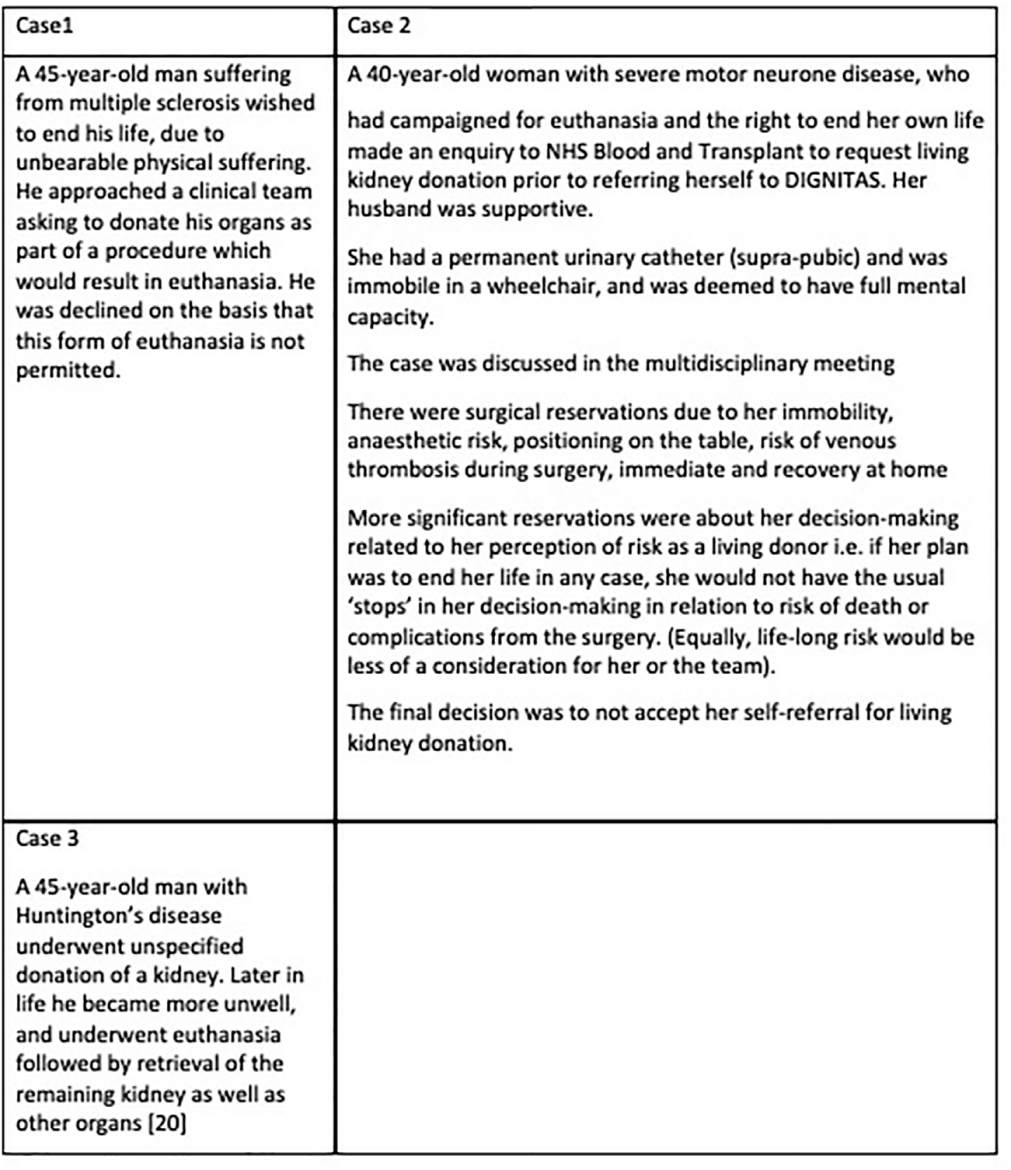
Examples of living donor candidates in the context of euthanasia.

Some potential donors might have a limited life expectancy, for example Huntington’s chorea, or a reduced capacity due to illness, for example, early dementia, but still wish to donate. In these cases, it might be argued that if the organ is unaffected by the underlying medical condition, donation does not hasten death, and there is sufficient capacity to make the decision, it would be reasonable to proceed (25). However other donors might wish to take more extreme risks-for example, donating their heart and thus ending their life (26–28). Similarly, there are those who are undergoing euthanasia (28), and wish to donate as part of that process, as detailed in Figure 4. In this case, the acceptance of such a donor would potentially help a number of recipients to have a better quality and quantity of life. However, apart from the fact that it is not permitted, such a procedure might have very negative consequences on wider donation rates, as the perception could be that life may be ended specifically to provide organs-a concern that has been expressed in general by some who are reluctant to agree to deceased donation. The principle that individuals are entitled to decide how and when they will die has been established in some countries (Switzerland), but some may struggle with the idea that doctors should participate in organ donation which might either precipitate death or be part of the final interventions.

## Conclusion

Decision making in the case of living donation remains difficult. There is a lack of detailed objective data regarding the risks in donors with co-morbidities, and the impact on the recipient of not proceeding. There are a number of potentially competing interests, including donor autonomy, the effect on the clinical team and wider societal effects on donation rates. One solution would be to introduce oversight removed from the clinical centre, or to designate some centres as those for “high risk” donors. Consideration of the understanding of risk by the donor may also help guide decisions. This manuscript provides an overview of the relevant issues for a clinical audience, and does not attempt a detailed ethical analysis, which is available in the bioethical literature; we have suggested further reading in Figure 2.

## Abbreviations

ELPAT, ethical legal and psychological aspects of transplantation; MDM, multidisciplinary meeting; ESRD, end stage renal disease; LDN, living donor nephrectomy.
